# Risk factors associated with hepatitis C virus infection: population-based cross-sectional study, Paraná, 2007–2022

**DOI:** 10.1590/S2237-96222026v35e20250196.en

**Published:** 2026-04-10

**Authors:** Carla Fernanda Tiroli, Alessandro Rolim Scholze, Gabriela Tavares Magnabosco, Maynara Fernanda Carvalho Barreto, Natacha Bolorino, Robson Cristiano Zandomenighi, Flávia Meneguetti Pieri

**Affiliations:** 1 Universidade Estadual de Londrina, Programa de Pós-Graduação em Enfermagem, Londrina, PR, Brazil; 2 Universidade Estadual de Maringá, Programa de Pós-Graduação em Enfermagem, Maringá, PR, Brazil

**Keywords:** Hepatitis C, Hepatitis, Viral, Human, Epidemiology, Disease Notification, Cross-Sectional Studies, Hepatitis C, Hepatitis Viral Humana, Epidemiología, Notificación de Enfermedades, Estudios Transversales

## Abstract

**Objective:**

To analyze the association between hepatitis C virus antibody (anti-HCV) results and demographic characteristics and exposure categories among reported cases in adult individuals in municipalities of Paraná.

**Methods:**

An analytical cross-sectional study was conducted using records from the Notifiable Diseases Information System (Sinan) for viral hepatitis. Analyses were performed using logistic regression. The lowest Akaike information criterion value was considered for the final model.

**Results:**

were presented as prevalence ratios (PR) with 95% confidence intervals (95%CI), and p-values <0.05 were considered significant.

**Results:**

The sample consisted of 32,829 cases, of which 35.4% had reactive anti-HCV results. The variables that showed statistically significant associations were: injecting drug use (PR 1.26; 95%CI 1.21; 1.33), blood transfusion (PR 1.20; 95%CI 1.16; 1.25), inhaled drug use (PR 1.13; 95% CI 1.09; 1.18), tattooing and piercing (PR 1.05; 95%CI 1.02; 1.08), and surgical treatment (PR 1.03; 95%CI 1.01; 1.05). Among protective factors, the following stood out: age group (PR 0.94; 95%CI 0.91; 0.96) and educational level (PR 0.97; 95%CI 0.95; 0.98).

**Conclusion:**

An association of anti-HCV with injecting and inhaled drug use, tattooing/piercing, and surgical treatment was evidenced. The findings reinforce the importance of early detection and risk stratification in addressing hepatitis C.

## Resumen

**Objetivo:**

Analizar la asociación entre los resultados del anticuerpo contra el virus de la hepatitis C (anti-VHC) y las características demográficas, así como las categorías de exposición, en casos notificados en personas adultas de los municipios de Paraná.

**Métodos:**

Estudio transversal analítico, realizado a partir de los registros del Sistema de Información de Agravamientos de Notificación (Sinan) de hepatitis virales. Los análisis se realizaron mediante regresión logística. Se consideró para el modelo final el menor valor del criterio de información de Akaike. Los resultados se presentaron como razón de prevalencia (RP), con intervalo de confianza del 95% (IC95%), y se adoptaron valores de p<0,05.

**Resultados:**

La muestra estuvo compuesta por 32.829 casos, de los cuales el 35,4% presentó reactividad anti-VHC. Las variables que mostraron una asociación estadísticamente significativa fueron: consumo de drogas inyectables (RP 1,26; IC95% 1,21; 1,33), transfusional (RP 1,20; IC95% 1,16; 1,25), drogas inhalables (RP 1,13; IC95% 1,09; 1,18), tatuajes y piercings (RP 1,05; IC95% 1,02; 1,08) y tratamiento quirúrgico (RP 1,03; IC95% 1,01; 1,05). Como factores de protección, destacaron el grupo etario (RP 0,94; IC95% 0,91; 0,96) y la escolaridad (RP 0,97; IC95% 0,95; 0,98).

**Conclusión:**

Se evidenció una asociación del anti-VHC con drogas inyectables e inhalables, tatuajes y piercings, y tratamiento quirúrgico. Los hallazgos refuerzan la importancia de la detección temprana y la estratificación del riesgo en el abordaje de la hepatitis C.

**Ethical aspects**

This research respected ethical principles, having obtained the following approval data:

Research ethics committee: Universidade Estadual de Londrina

Opinion number: 6,328,274

Approval date: 27/9/2023

Certificate of submission for ethical appraisal: 73429023.6.0000.5231

Informed consent form: Exempt.

## Introduction 

Hepatitis C was responsible for 17% of the 1.3 million global deaths from viral hepatitis in 2022, according to the World Health Organization (WHO). In Brazil, between 2000 and 2024, more than 342 thousand cases were confirmed. The adoption of more sensitive diagnostic criteria from 2015 onward increased detection rates, which reached 12.2 per 100 thousand inhabitants in 2018, followed by a subsequent decrease (1). During the same period, in 2018, Paraná recorded 2,771 deaths from the hepatitis C virus (2,3). 

In 2016, the World Health Organization (WHO) established targets to eliminate hepatitis C as a public health threat by 2030, aiming to diagnose 90% of cases, treat 80% of diagnosed individuals, and achieve a 65% reduction in disease-related mortality (4,5). Aligned with this target, in 2024, the Brazilian government launched the Healthy Brazil Program- Unite to Care (*Programa Brasil Saudável* – *Unir para Cuidar*), which strengthens surveillance, expands access to care, and enhances primary health care interventions, particularly in territories with a high burden of viral hepatitis (5,6).

Although viral hepatitis types share similar clinical and epidemiological characteristics, each has distinct features that affect the effectiveness of control measures (7). Identifying factors associated with hepatitis C virus infection and mapping the demographic and behavioral profiles of affected populations are essential steps to support strategies aligned with the guidelines of the Healthy Brazil Program and the Sustainable Development Goals (SDG), which aim to ensure healthy lives and promote well-being at all ages (1,5). 

This study aimed to analyze the association between hepatitis C virus antibody (anti-HCV) results and demographic characteristics and exposure categories among reported adult cases in municipalities of Paraná. It sought to contribute to the implementation of actions recommended by the Healthy Brazil Program. 

## Methods 

### Study design and data source

This is an observational, cross-sectional (8), population-based study conducted using secondary data from viral hepatitis notification forms recorded in the Notifiable Diseases Information System (*Sistema de Informação de Agravos de Notificação*, Sinan), made available by the São Paulo State Health Department in a Microsoft Excel spreadsheet, version 16.0, on 27 September 2023. The research objective justifies the choice of a cross-sectional design. The study manuscript followed the recommendations of the Strengthening the Reporting of Observational Studies in Epidemiology (STROBE) checklist (9).

### Setting 

The study was conducted in Paraná, located in the Southern region of Brazil, with 399 municipalities, an area of 199,298.981 km^2^, and an estimated population of 11,824,665 inhabitants, making it the fifth most populous state in the country in 2022 (10). The territory is divided into four health macroregions (East, North, Northwest, and West), subdivided into 22 Regional Health Offices (11).

### Participants and study size

All reported cases with results from rapid testing or anti-HCV serological markers (reactive or non-reactive), aged ≥18 years, residing in Paraná at the time of diagnosis, between 1 January 2007, and 31 December 2022, were included. Cases with missing data for exposure variables were excluded. No sample size calculation was performed, as the study used the total number of reported cases that met the inclusion criteria during the study period.

### Variables 

The study treated anti-HCV as the outcome variable, with participants categorized as reactive or non-reactive. Explanatory variables were categorized into demographic and exposure-related categories.

- Demographic: sex (female, male); age in years (18–59, ≥60); educational level in years of schooling (up to 9, ≥10 years); race/skin color (White, Non-white); and municipality size (small=up to 50,000 inhabitants; medium = 50,001–100,000 inhabitants; large = 100,001–900,000 inhabitants) (6).
- Exposure: reported types of exposure categories – dental treatment, injectable medication, surgical treatment, three or more partners, tattooing/piercing, inhaled drugs or crack, blood transfusion, acupuncture, hemodialysis, biological material, and transplantation. All exposure categories were dichotomized into yes/no.

### Bias and statistical methods 

Bivariate analysis was performed on sociodemographic and clinical-epidemiological characteristics, stratified by sex (male, female), using IBM® Statistical Package for the Social Sciences (IBM® SPSS), version 29.0.0 (12).

The remaining analyses were conducted in R, version 4.3.1, adopting p-values<0.05 to reject the null hypothesis, indicating that the predictor contributed significantly to the regression model.

The identification of explanatory variables (predictors) associated with anti-HCV was performed by fitting a generalized linear model with a Poisson family and a log link function, appropriate for estimating prevalence ratios (PR) for binary outcomes in cross-sectional studies. To ensure consistent estimates of standard errors, robust sandwich variance correction was applied.

The final regression model was selected as the one with the lowest Akaike information criterion (AIC) among the fits, using a forward-backward variable selection strategy. Although the AIC can be used to compare models, it is important to note that in Poisson models with robust variance, the AIC may not accurately reflect model fit, as the robust variance does not alter the log-likelihood used in the AIC calculation. Therefore, estimates of epidemiological plausibility and coefficient stability were also considered. 

Estimated coefficients (β) were exponentiated to obtain PR, allowing the interpretation of the relative effect of each explanatory variable on the prevalence of the outcome (reactive anti-HCV), adjusted for the other covariates. 95% confidence intervals (95%CI) were calculated (13).

Model fit was assessed through Pearson residual analysis, evaluation of influential values and leverage (Cook’s distance), and graphical assessment of residual linearity and variance. Residual analysis of the regression model is essential for evaluating the fit. For this purpose, randomized quantile residuals were calculated for the generalized linear models, followed by the construction of a quantile-quantile (Q-Q) plot to compare the observed quantiles with the theoretical quantiles of a normal distribution, thereby allowing assessment of residual normality. The asymptotic Kolmogorov–Smirnov test was also applied, along with descriptive presentation of the randomized quantile residuals (mean and variance) (13).

The final regression model included 13 explanatory variables: age group, years of schooling, race/skin color, injecting drug use, blood transfusion, inhaled drugs/crack, tattooing/piercing, surgical treatment, hemodialysis, injectable medications, biological material, transplantation, and three or more sexual partners.

As this study used secondary data, the main potential biases included: information bias, due to underreporting or inconsistencies in the completion of Notifiable Diseases Information System (Sinan) forms, particularly for exposure variables; selection bias, related to the exclusion of records with incomplete data; and measurement bias, since anti-HCV positivity may not indicate active infection but only prior contact with the virus.

To mitigate these biases, only records with complete information for the main variables were included, and robust statistical techniques for model adjustment and validation were applied.

## Results 

Of the 50,721 eligible records, 35.2% (17,892) were excluded due to missing exposure variable information, leaving 32,829 cases analyzed. Most cases were concentrated in large municipalities, particularly in the Regional Health Offices of Curitiba (32.0%), Londrina (10.1%), Maringá (8.2%), and Foz do Iguaçu (8.1%). A predominance of male sex was observed (55.7%), with a median age of 44 years (Table 1).

**Table 1:** Distribution of demographic characteristics and risk exposures by sex. Paraná, 2007–2022 (n=32,829)

Variable	Sex		Total
	Male (n=18.278)	Female (n=14.551)	
	n (%)	n (%)	
**Age group (years)**			
18-59	15.715 (55,7)	12.493 (44,3)	28.208
≥60	2.563 (55,5)	2.058 (44,5)	4.621
**Educational level (years of schooling)**			
Up to 9	10.367 (57,1)	7.794 (42,9)	18.161
≥10	7.911 (53,9)	6.757 (46,1)	14.668
**Race/skin color**			
White	14.207 (55,4)	11.453 (44,6)	25.660
Non-White	4.071 (56,8)	3.098 (43,2)	7.169
Anti-HCV^a^			
Reactive	6.794 (58,4)	4.833 (41,6)	11.627
Non-reactive	11.484 (54,2)	9.718 (45,8)	21.202
**Municipality Size**			
Small	5.485 (53,0)	4.864 (47,0)	10.349
Medium	1.882 (51,8)	1.753 (48,2)	3.635
Large	10.911 (57,9)	7.934 (42,1)	18.845
**Reported types of exposure categories ^a^**			
Dental treatment	7.170 (54,9)	5.894 (45,1)	13.064
Injectable medication	5.853 (54,5)	4.885 (45,5)	10.738
Surgical treatment	4.583 (49,6)	4.662 (50,4)	9.245
Three or more partners	4.665 (71,5)	1.863 (28,5)	6.528
Tattoing/piercing	2.704 (60,0)	1.804 (40,0)	4.508
Inhaled drugs or crack	2.560 (81,2)	592 (18,8)	3.152
Injectable drugs	1.579 (80,9)	373 (19,1)	1.952
Transfusion	1.173 (47,6)	1.291 (52,4)	2.464
Acupuncture	513 (46,1)	601 (53,9)	1.114
Hemodialysis	345 (57,5)	255 (42,5)	600
Biological material	201 (32,6)	416 (67,4)	617
Transplantation	191 (60,8)	123 (39,2)	314

^a^Anti-HCV: antibody against hepatitis C virus; ^b^Percentages do not sum to 100.0% because each participant could select more than one option

Statistically significant associations were observed between exposure to injecting drugs and reactive anti-HCV (PR 1.26; 95%CI 1.21; 1.33), followed by blood transfusion (1.20) and inhaled drugs/crack use (1.13). Protective factors included age group (PR 0.94; 95%CI 0.91; 0.96) and years of schooling (PR 0.97; 95%CI 0.95; 0.98) (Table 2). 

**Table 2:** Prevalence ratio (PR), 95% confidence interval (95%CI), and p-value from logistic regression adjusted for explanatory variables between reactive hepatitis C virus antibody and demographic and exposure variables. Paraná, 2007–2022 (n=32,829)

Explanatory variables	Reactive n (%)	Non-reactive n (%)	Total	PR (95%CI)	p-value
**Demographic variables**					
**Age group (years)**					
18-59	9.553 (33,9)	18.655 (66,1)	28.208	0,94 (0,91; 0,96)	<0,001
≥60	1.995 (43,2)	2.626 (56,8)	4.621	1,00	
**Race/skin color**					
White	8.812 (34,3)	16.848 (65,7)	25.660	0,98 (0,96; 1,01)	0,091
Non-White	2.736 (38,2)	4.433 (61,8)	7.169	1,00	
**Educational level (years of schooling**					
Up to 9	6.189 (34,1)	11.972 (65,9)	18.161	0,97 (0,95; 0,98)	<0,001
≥10	5.359 (36,5)	9.309 (63,5)	14.668	1,00	
**Exposure variables**					
Injectable medication	4.036 (37,6)	6.702 (62,4)	10.738	0,98 (0,97; 1,10)	0,160
Surgical treatment	3.958 (42,8)	5.287 (57,2)	9.245	1,03 (1,01; 1,05)	<0,001
Three or more partners	2.675 (41,0)	3.853 (59,0)	6.528	0,99 (0,97; 1,01)	0,325
Tattooing and piercing	2.283 (50,6)	2.225 (49,4)	4.508	1,05 (1,02; 1,08)	<0,001
Inhaled drugs	2.152 (68,3)	1.000 (31,7)	3.152	1,13 (1,09; 1,18)	<0,001
Transfusion	1.589 (64,5)	875 (35,5)	2.464	1,20 (1,16; 1,25)	<0,001
Injectable drugs	1.594 (81,7)	358 (18,3)	1.952	1,26 (1,21; 1,33)	<0,001
Biological material	246 (39,9)	371 (60,1)	617	0,95 (0,89; 1,01)	0,120
Hemodialysis	336 (56,0)	264 (44,0)	600	1,04 (0,96; 1,12)	0,360
Transplantation	192 (61,1)	122 (38,9)	314	0,95 (0,85; 1,06)	0,353

## Discussion 

The findings of this study highlighted factors associated with hepatitis C virus infection in Paraná, providing evidence to support the enhancement of actions recommended by the Healthy Brazil Program. The identified associations can guide prevention and control strategies, particularly among vulnerable populations, contributing to the reduction of inequities and to addressing socially determined health conditions (14).

Identifying risk behaviors and exposure categories enables the development of health education strategies tailored to different population profiles. These actions strengthen care coordination and intersectoral practices, contributing to the achievement of targets to eliminate hepatitis C as a public health problem by 2030 (14).

Primary health care, as recommended by the Brazilian Unified Health System (Sistema *Único de Saúde*, SUS), is responsible for health promotion, prevention, diagnosis, and timely referral to specialized care (15). Therefore, the higher concentration of cases in large municipalities, such as Curitiba, Londrina, Maringá, and Foz do Iguaçu, may result from broader access to health services and the decentralization of rapid testing. 

The Paraná State Health Department, in accordance with the Ministry of Health guidelines, emphasizes the importance of strengthening the viral hepatitis care line, including rapid testing, educational campaigns, and coordination with specialized services (16).

Injecting drug use showed the highest prevalence ratio for reactive anti-HCV, followed by blood transfusion and inhaled drugs or crack use. Direct percutaneous exposure to blood is recognized as the main route of transmission among drug users. In Brazil, more than 1.4 million people reported using these substances, making this group a priority for harm reduction interventions, such as provision of disposable supplies, educational interventions, and access to medications like partial opioid receptor agonists (14,17–18).

The study results indicate that the spread of hepatitis C does not occur homogeneously across the population, as economic, social, and environmental factors influence it. The impact of economic factors can be inferred from the higher vulnerability of certain population groups, which face structural barriers that increase infection risk, even though the study did not directly evaluate indicators such as income or access to health services. Social factors, such as greater exposure among vulnerable groups (e.g., drug users), and environmental factors, such as inadequate health service infrastructure, may also facilitate virus transmission. Recognizing these inequalities, the creation of the Interministerial Committee for the Elimination of Tuberculosis and Other Socially Determined Diseases (*Comitê Interministerial para a Eliminação da Tuberculose e de Outras Doenças Determinadas Socialmente*, CIEDDS), which includes viral hepatitis, represents progress in intersectoral coordination of public policies focused on social determinants (14,19).

Protective factors included age group and educational level. Younger individuals tend to have greater access to information, higher adherence to preventive practices, and lower exposure to risk behaviors associated with infection. Although there was a predominance of individuals with up to nine years of schooling, this characteristic did not constitute a risk factor for hepatitis C transmission in the studied population. This may be due to specific social context and access to health services (1,20).

Despite efforts, challenges remain in implementing and enforcing these policies effectively. Practical application of the guidelines depends on the integration of managers, professionals, and health service users. Proposed actions must translate into concrete interventions contextualized to the areas.

The results of this study allowed the identification of demographic factors and exposure categories associated with reactive anti-HCV among adults reported in Paraná, with particular emphasis on injecting drug use, blood transfusion, inhaled drugs/crack, tattooing/piercing, and surgical treatment. Age group and educational level emerged as protective factors. These findings addressed the study objective and highlighted relevant epidemiological patterns, thereby strengthening the actions outlined in the Healthy Brazil Program. This helps identify priority groups and guide strategies for prevention, early diagnosis, and continuous care. 

## References

[1] Brasil. Ministério da Saúde. (2025 ). Governo Federal avança nas ações para eliminação das hepatites virais no país [Internet].

[2] World Health Organization (2024 ). Hepatitis C [Internet].

[3] Brasil. Ministério da Saúde. (2024 ). Boletim Epidemiológico - hepatites virais 2024.

[4] World Health Organization (2022 ). Updated recommendations on treatment of adolescents and children with chronic HCV infection, and HCV simplified service delivery and diagnostics [Internet].

[5] Brasil. Ministério da Saúde. (2018 ). Manual técnico para o diagnóstico das hepatites virais.

[6] Instituto Brasileiro de Geografia e Estatística (2022 ). Panorama 2022 [Internet].

[7] Brasil. Presidência da República. (2024). Decreto nº 11.908, de 6 de fevereiro de 2024. Institui o Programa Brasil Saudável - Unir para Cuidar, e altera o Decreto nº 11.494, de 17 de abril de 2023, para dispor sobre o Comitê Interministerial para a Eliminação da Tuberculose e de Outras Doenças Determinadas Socialmente - CIEDDS.

[8] Andrade SM, Mesas AE, González AD, Girotto E (2017). Bases da saúde coletiva.

[9] Von Elm E, Altman DG, Egger M, Pocock SJ, Gøtzsche PC, Vandenbroucke JP (2008). Strengthening the reporting of observational studies in epidemiology (STROBE) statement: guidelines for reporting observational studies. J Clin Epidemiol.

[10] Instituto Brasileiro de Geografia e Estatística (2023 ). Censo demográfico 2022: população e densidade demográfica do Paraná [Internet].

[11] Paraná (2023 ). Secretaria de Estado da Saúde do Paraná.

[12] IBM (2024 ). Downloading IBM SPSS Statistics 29 [Internet].

[13] Hilbe JM (2015). Practical guide to logistic regression.

[14] Brasil. Ministério da Saúde. (2025). Diretrizes Nacionais do Programa Brasil Saudável: unir para cuidar.

[15] Brasil (2018 ). Ministério da Saúde.

[16] Paraná. Secretaria de Saúde do Paraná. (2017 ). Linha de cuidado hepatites virais [Internet].

[17] Nações Unidas Brasil (2020 ). Número de pessoas que usaram drogas em 2020 é 26% maior do que em 2010 [Internet].

[18] Organização das Nações Unidas. (2023 ). ONU quer prevenir contaminação de hepatite C entre usuários de drogas injetáveis [Internet].

[19] Brasil (2023). Presidência da República.

[20] Dai S, Wang Z, Guo Q, Tang G, Guo Q, Zhang J, Fan Y (2025). Awareness of hepatitis C prevention and treatment and highrisk behaviors among the general population in Anhui Province: a crosssectional study. Front Public Health.

