# Supplementary material for: Risk factors associated with hepatitis C virus infection: population-based cross-sectional study, Paraná, 2007–2022
Source: Epidemiol Serv Saude. 2026 Apr 10;35:e20250196. doi: 10.1590/S2237-96222026v35e20250196.en (PMC13073088; doi:10.1590/S2237-96222026v35e20250196.en)
Supplement: Translation

# Translation: Fatores de risco associados à infecção pelo vírus da hepatite C: estudo transversal de base populacional, Paraná, 2007-2022

**DOI:** 10.1590/S2237-96222026v35e20250196.pt

**Authors:** Carla Fernanda Tiroli, Alessandro Rolim Scholze, Gabriela Tavares Magnabosco, Maynara Fernanda Carvalho Barreto, Natacha Bolorino, Robson Cristiano Zandomenighi, Flávia Meneguetti Pieri

**Affiliations:** 1 Universidade Estadual de Londrina, Programa de Pós-Graduação em Enfermagem, Londrina, PR, Brasil; 2 Universidade Estadual de Maringá, Programa de Pós-Graduação em Enfermagem, Maringá, PR, Brasil

**Keywords:** Hepatite C, Hepatite Viral Humana, Epidemiologia, Notificação de Doença, Estudos Transversais

## Abstract

**Objetivo:**

Analisar a associação dos resultados do anticorpo contra o vírus da hepatite C (anti-HCV) com as características demográficas e as categorias de exposição entre os casos notificados em pessoas adultas nos municípios do Paraná.

**Métodos:**

Estudo transversal analítico, realizado a partir das fichas do Sistema de Informação de Agravos de Notificação de hepatites virais. As análises foram calculadas por meio de regressão logística. O menor valor de critério de informação de Akaike foi considerado para o modelo final. Os resultados foram apresentados em forma de razão de prevalência (RP), com intervalo de confiança de 95% (IC95%), sendo adotados os p-valores inferiores a 0,05.

**Resultados:**

A amostra foi composta de 32.829 casos, dos quais 35,4% tiveram o anti-HCV reagente. As variáveis que apresentaram associação estatisticamente significativa foram: drogas injetáveis (RP 1,26; IC95% 1,21; 1,33), transfusional (RP 1,20; IC95% 1,16; 1,25), drogas inaláveis (RP 1,13; IC95% 1,09; 1,18), tatuagem e piercing (RP 1,05; IC95% 1,02; 1,08), tratamento cirúrgico (RP 1,03; IC95% 1,01; 1,05). Como fatores de proteção, destacam-se: faixa etária (RP 0,94; IC95% 0,91; 0,96) e escolaridade (RP 0,97; IC95% 0,95; 0,98).

**Conclusão:**

Evidenciou-se associação do anti-HCV com drogas injetáveis, inaláveis, tatuagem/piercing e tratamento cirúrgico. Os achados reforçam a importância de detectar precocemente e estratificar o risco no enfrentamento à hepatite C.

**Aspectos éticos**

Esta pesquisa respeitou os princípios éticos, obtendo os seguintes dados de aprovação:

Comitê de ética em pesquisa: Universidade Estadual de Londrina

Número do parecer: 6.328.274

Data de aprovação: 27/9/2023

Certificado de apresentação de apreciação ética: 73429023.6.0000.5231

Registro do consentimento livre e esclarecido: Dispensado.

## Introdução 

A hepatite C foi responsável por 17% dos 1,3 milhão de óbitos globais por hepatites virais em 2022, segundo a Organização Mundial da Saúde. No Brasil, entre 2000 e 2024, foram confirmados mais de 342 mil casos. A adoção de critérios diagnósticos mais sensíveis a partir de 2015 elevou as taxas de detecção, que atingiram 12,2 por 100 mil habitantes em 2018, com posterior queda (1). No mesmo período, em 2018, o Paraná registrou 2.771 mortes por vírus da hepatite C (2,3). 

A Organização Mundial da Saúde estabeleceu, em 2016, metas para eliminar a hepatite C como ameaça à saúde pública até 2030, prevendo o diagnóstico de 90% dos casos, o tratamento de 80% das pessoas diagnosticadas e a redução de 65% na mortalidade pela doença (4,5). Alinhado a essa meta, em 2024, o governo brasileiro criou o Programa Brasil Saudável, que fortalece a vigilância, amplia o acesso ao cuidado e aprimora as ações na atenção primária à saúde, especialmente em territórios com alta carga de hepatites virais (5,6).

Embora as hepatites virais compartilhem características clínicas e epidemiológicas semelhantes, cada tipo apresenta particularidades que impactam a efetividade das ações de controle (7). Identificar fatores associados à infecção pelo vírus da hepatite C e traçar os perfis demográfico e comportamental da população acometida são etapas fundamentais para subsidiar estratégias alinhadas às diretrizes do Programa Brasil Saudável – Unir para Cuidar e aos objetivos de desenvolvimento sustentável, que visam assegurar vidas saudáveis e promover o bem-estar em todas as idades (1,5). 

Este estudo teve como objetivo analisar a associação dos resultados do anticorpo contra o vírus da hepatite C (anti-HCV) com as características demográficas e as categorias de exposição entre os casos notificados em pessoas adultas nos municípios do Paraná. Visou-se contribuir para a implementação das ações preconizadas pelo Programa Brasil Saudável. 

## Métodos 

### Delineamento do estudo e fonte de dados

Trata-se de estudo observacional, do tipo transversal (8) de base populacional, desenvolvido com dados secundários provenientes das fichas de notificação de hepatites virais registradas no Sistema de Informação de Agravos de Notificação, disponibilizados pela Secretaria de Estado da Saúde do Paraná em planilha do Microsoft Excel, na versão 16.0, em 27 de setembro de 2023. A escolha pelo delineamento transversal justifica-se pelo objetivo desta pesquisa. A redação do estudo seguiu as recomendações do checklist Strengthening the Reporting of Observational Studies in Epidemiology (9).

### Contexto 

O estudo foi conduzido no Paraná, localizado na região Sul do Brasil, com 399 municípios, área de 199.298.981 km^2^ e população estimada em 11.824.665 habitantes, sendo o quinto estado mais populoso do país em 2022 (10). O território é dividido em quatro macrorregionais de saúde (Leste, Norte, Noroeste e Oeste), subdivididas em 22 regionais de saúde (11).

### Participantes e tamanho do estudo

Foram incluídos todos os casos notificados com resultado do teste rápido ou marcador sorológico anti-HCV (reagente ou não reagente), com idade ≥18 anos, residentes no Paraná no momento do diagnóstico, entre 1º de janeiro de 2007 e 31 de dezembro de 2022. Casos em que não houve preenchimento das variáveis de exposição foram excluídos. Não foi realizado cálculo amostral, uma vez que se utilizou o total de casos notificados que atenderam aos critérios de inclusão no período.

### Variáveis 

O estudo foi realizado considerando como variável desfecho o anti-HCV, o qual foi categorizado como reagente e não reagente. As variáveis explicativas foram categorizadas como demográficas e de exposição.

- Demográficas: sexo (feminino, masculino); idade em anos (18-59, ≥60); escolaridade em anos de estudo (até 9, ≥10 anos); raça/cor da pele (branca, não branca); e porte dos municípios (pequeno porte=até 50.000 habitantes; médio porte=50.001-100.000 habitantes; e grande porte=100.001-900.000 habitantes) (6).
- De exposição: relato dos tipos de categorias de exposição – tratamento dentário, medicamentos injetáveis, tratamento cirúrgico, três ou mais parceiros, tatuagem/piercing, drogas inaláveis ou crack, transfusional, acupuntura, hemodiálise, material biológico e transplante. Todas essas categorias foram dicotomizadas em sim e não.

### Viés e métodos estatísticos 

Foi realizada análise bivariada com as características sociodemográficas e clínico-epidemiológicas, segundo variáveis do sexo (masculino, feminino), no programa IBM Statistical Package for the Social Sciences, versão 29.0.0 (12).

O restante das análises foi realizado no programa estatístico R, versão 4.3.1, adotando os p-valores inferiores a 0,05 para rejeitar a hipótese nula, isto é, o preditor contribuiu significativamente para o modelo de regressão.

A identificação das variáveis explicativas (preditores) associadas ao anti-HCV ocorreu por meio da elaboração de um modelo linear generalizado com família Poisson e função de ligação log, adequado para estimar razões de prevalência (RP) em desfechos binários de estudos transversais. Para assegurar estimativas consistentes dos erros-padrão, foi aplicada a correção por variância robusta do tipo sandwich.

O modelo de regressão final foi considerado o menor valor de critério de informação de Akaike do respectivo ajuste, tendo como estratégia de inserção das variáveis o método forward-backward de seleção. Embora o critério de informação de Akaike possa ser utilizado para comparar modelos, é importante observar que, em modelos Poisson com variância robusta, o critério de informação de Akaike pode não refletir corretamente o ajuste do modelo, pois a variância robusta não altera a log-verossimilhança usada no critério de informação de Akaike. Portanto, foram também consideradas as estimativas de plausibilidade epidemiológica e estabilidade dos coeficientes. 

Os coeficientes estimados (β) foram exponenciados para obter as RP, permitindo a interpretação do efeito relativo de cada variável explicativa sobre a prevalência do desfecho (anti-HCV reagente), ajustada pelas demais covariáveis. Foram calculados os valores de intervalo de confiança em 95% (IC95%) (13).

A avaliação do ajuste do modelo foi realizada por meio da análise de resíduos de Pearson, verificação de valores influentes e alavancagem (*Cook’s distance*), bem como análise gráfica da linearidade e variância dos resíduos. A análise dos resíduos do modelo de regressão é essencial para identificar como foi o ajuste. Para isso, foram calculados os resíduos quantílicos aleatórios para modelos lineares generalizados e, em seguida, construído um gráfico quantil-quantil (QQ plot) com a finalidade de comparar os quantis dos resíduos observados com os quantis teóricos da distribuição normal, permitindo avaliar a normalidade dos resíduos, foi utilizado o teste assintótico de Kolmogorov-Smirnov, além da apresentação descritiva dos resíduos quantílicos randomizados (média e variância) (13).

O modelo de regressão final foi composto de 13 variáveis explicativas: faixa etária, escolaridade, raça/cor da pele, drogas injetáveis, transfusional, drogas inaláveis/crack, tatuagem/piercing, tratamento cirúrgico, hemodiálise, medicamentos injetáveis, material biológico, transplante e três ou mais parceiros.

Por se tratar de estudo com dados secundários, os principais potenciais vieses incluíram: viés de informação, decorrente de subnotificação ou inconsistência no preenchimento das fichas do Sistema de Informação de Agravos de Notificação, especialmente em variáveis de exposição; viés de seleção, relacionado à exclusão de registros com dados incompletos; e viés de mensuração, pois o resultado anti-HCV pode não significar infecção ativa, mas apenas contato prévio com o vírus.

Para mitigar esses vieses, foram incluídos apenas registros com informações completas nas variáveis principais e aplicadas técnicas estatísticas robustas de ajuste e validação dos modelos.

## Resultados 

Dos 50.721 registros elegíveis, 35,2% (17.892) foram excluídos devido à informação ausente nas variáveis de exposição, o que resultou em 32.829 casos analisados. A maioria concentrava-se em municípios de grande porte, sobretudo nas regionais de saúde de Curitiba (32,0%), Londrina (10,1%), Maringá (8,2%) e Foz do Iguaçu (8,1%). Observou-se predominância do sexo masculino (55,7%), com mediana de idade de 44 anos (Tabela 1).

**Tabela 1:** Distribuição das características demográficas e exposição de risco segundo o sexo. Paraná, 2007-2022 (n=32.829)

Variável	Sexo		Total
	Masculino (n=18.278)	Feminino (n=14.551)	
	n (%)	n (%)	
**Faixa etária** (anos)			
18-59	15.715 (55,7)	12.493 (44,3)	28.208
≥60	2.563 (55,5)	2.058 (44,5)	4.621
**Escolaridade** (anos)			
Até 9	10.367 (57,1)	7.794 (42,9)	18.161
≥10	7.911 (53,9)	6.757 (46,1)	14.668
**Raça/cor da pele**			
Branca	14.207 (55,4)	11.453 (44,6)	25.660
Não branca	4.071 (56,8)	3.098 (43,2)	7.169
Anti-HCV^a^			
Reagente	6.794 (58,4)	4.833 (41,6)	11.627
Não reagente	11.484 (54,2)	9.718 (45,8)	21.202
**Porte dos municípios**			
Pequeno	5.485 (53,0)	4.864 (47,0)	10.349
Médio	1.882 (51,8)	1.753 (48,2)	3.635
Grande	10.911 (57,9)	7.934 (42,1)	18.845
**Relato dos tipos de categorias de exposição^a^**			
Tratamento dentário	7.170 (54,9)	5.894 (45,1)	13.064
Medicamentos injetáveis	5.853 (54,5)	4.885 (45,5)	10.738
Tratamento cirúrgico	4.583 (49,6)	4.662 (50,4)	9.245
Três ou mais parceiros	4.665 (71,5)	1.863 (28,5)	6.528
Tatuagem/piercing	2.704 (60,0)	1.804 (40,0)	4.508
Drogas inaláveis ou crack	2.560 (81,2)	592 (18,8)	3.152
Drogas injetáveis	1.579 (80,9)	373 (19,1)	1.952
Transfusional	1.173 (47,6)	1.291 (52,4)	2.464
Acupuntura	513 (46,1)	601 (53,9)	1.114
Hemodiálise	345 (57,5)	255 (42,5)	600
Material biológico	201 (32,6)	416 (67,4)	617
Transplante	191 (60,8)	123 (39,2)	314

^a^Anti-HCV: anticorpo contra o vírus da hepatite C; ^b^As porcentagens não somam 100,0% porque cada voluntário poderia marcar várias opções.

Verificou-se a associação estatisticamente significativa entre a exposição a drogas injetáveis e o anti-HCV reagente (RP 1,26; IC95% 1,21; 1,33), seguida por transfusão (1,20) e uso de drogas inaláveis/crack (1,13). Como fatores de proteção, destacaram-se a faixa etária (0,94; IC95% 0,91; 0,96) e a escolaridade (0,97; IC95% 0,95; 0,98) (Tabela 2). 

**Tabela 2:** Razão de prevalência (RP), intervalo de confiança de 95% (IC95%) e p-valor da regressão logística ajustada às variáveis explicativas entre anticorpo contra o vírus da hepatite C reagente e variáveis demográficas e de exposição. Paraná, 2007-2022 (n=32.829)

Variáveis explicativas	Reagente n (%)	Não reagente n (%)	Total	RP (IC95%)	p-valor
**Variáveis demográficas**					
**Faixa etária** (anos)					
18-59	9.553 (33,9)	18.655 (66,1)	28.208	0,94 (0,91; 0,96)	<0,001
≥60	1.995 (43,2)	2.626 (56,8)	4.621	1,00	
**Raça/cor da pele**					
Branca	8.812 (34,3)	16.848 (65,7)	25.660	0,98 (0,96; 1,01)	0,091
Não branca	2.736 (38,2)	4.433 (61,8)	7.169	1,00	
**Escolaridade** (anos)					
Até 9	6.189 (34,1)	11.972 (65,9)	18.161	0,97 (0,95; 0,98)	<0,001
≥10	5.359 (36,5)	9.309 (63,5)	14.668	1,00	
**Variáveis de exposição**					
Medicamentos injetáveis	4.036 (37,6)	6.702 (62,4)	10.738	0,98 (0,97; 1,10)	0,160
Tratamento cirúrgico	3.958 (42,8)	5.287 (57,2)	9.245	1,03 (1,01; 1,05)	<0,001
Três ou mais parceiros	2.675 (41,0)	3.853 (59,0)	6.528	0,99 (0,97; 1,01)	0,325
Tatuagem e piercing	2.283 (50,6)	2.225 (49,4)	4.508	1,05 (1,02; 1,08)	<0,001
Drogas inaláveis	2.152 (68,3)	1.000 (31,7)	3.152	1,13 (1,09; 1,18)	<0,001
Transfusional	1.589 (64,5)	875 (35,5)	2.464	1,20 (1,16; 1,25)	<0,001
Drogas injetáveis	1.594 (81,7)	358 (18,3)	1.952	1,26 (1,21; 1,33)	<0,001
Material biológico	246 (39,9)	371 (60,1)	617	0,95 (0,89; 1,01)	0,120
Hemodiálise	336 (56,0)	264 (44,0)	600	1,04 (0,96; 1,12)	0,360
Transplante	192 (61,1)	122 (38,9)	314	0,95 (0,85; 1,06)	0,353

## Discussão 

Os achados deste estudo evidenciaram fatores associados à infecção pelo vírus da hepatite C no Paraná, oferecendo subsídios para aprimorar as ações recomendadas pelo Programa Brasil Saudável. As associações identificadas podem orientar estratégias de prevenção e de controle, especialmente em populações vulneráveis, o que contribui para a redução de iniquidades e para o enfrentamento das doenças determinadas socialmente (14).

Identificar comportamentos de risco e categorias de exposição possibilita o desenvolvimento de estratégias de educação em saúde voltadas aos diferentes perfis populacionais. Essas ações fortalecem a coordenação do cuidado e as práticas intersetoriais, o que contribui para o alcance das metas de eliminação da hepatite C como problema de saúde pública até 2030 (14).

A atenção primária à saúde, conforme o Sistema Único de Saúde preconiza, é responsável pela promoção da saúde, da prevenção, do diagnóstico e do encaminhamento oportuno para o cuidado especializado (15). Sendo assim, a maior concentração de casos em municípios de grande porte, como Curitiba, Londrina, Maringá e Foz do Iguaçu, pode ser resultado de acesso mais amplo aos serviços de saúde e à descentralização do teste rápido. 

A Secretaria de Estado da Saúde do Paraná, em consonância com as diretrizes do Ministério da Saúde, reforça a importância do fortalecimento da linha de cuidado das hepatites virais, com testagem rápida, campanhas educativas e articulação com serviços especializados (16).

O uso de drogas injetáveis apresentou a maior razão de prevalência para anti-HCV reagente, seguido pelo transfusional e uso de drogas inaláveis ou crack. A exposição percutânea direta ao sangue é reconhecida como via principal de transmissão entre usuários de drogas. No Brasil, mais de 1,4 milhão de pessoas relataram uso dessas substâncias, o que configura grupo prioritário para ações de redução de danos, como oferta de insumos descartáveis, intervenções educativas e acesso a medicamentos como os agonistas parciais de receptores opioides (14,17-18).

Os resultados do estudo indicam que a disseminação da hepatite C não ocorre de maneira uniforme na população, pois está condicionada a fatores econômicos, sociais e ambientais. A influência de fatores econômicos pode ser inferida a partir da maior vulnerabilidade de determinados grupos populacionais, que enfrentam barreiras estruturais capazes de aumentar o risco de infecção, mesmo que o estudo não tenha avaliado diretamente indicadores como renda ou acesso a serviços de saúde. Fatores sociais, como a maior exposição de grupos vulneráveis, a exemplo de usuários de drogas, e fatores ambientais, como a precariedade na infraestrutura dos serviços de saúde, também podem favorecer a transmissão do vírus. Reconhecendo-se essas desigualdades, a criação do Comitê Interministerial para a Eliminação da Tuberculose e de Outras Doenças Determinadas Socialmente (CIEDDS), que inclui as hepatites virais, representa um avanço na articulação intersetorial de políticas públicas com foco nos determinantes sociais (14,19).

Como fatores de proteção, destacaram-se a faixa etária e o nível de escolaridade. Indivíduos mais jovens tendem a apresentar maior acesso à informação, maior adesão às práticas preventivas e menor exposição a comportamentos de risco associados à infecção. Embora tenha ocorrido predominância de indivíduos com até nove anos de escolaridade, essa característica não se configurou como fator de risco para a transmissão da hepatite C na população estudada. Possivelmente isso se dá por especificidades do contexto social e do acesso aos serviços de saúde (1,20).

Apesar dos esforços, persistem desafios na implementação e eficácia dessas políticas. A aplicação prática das diretrizes depende da integração entre gestores, profissionais e usuários dos serviços de saúde. É necessário que as ações propostas se transformem em intervenções concretas e contextualizadas nos territórios.

Os resultados deste estudo permitiram identificar fatores demográficos e categorias de exposição associados ao anti-HCV reagente entre adultos notificados no Paraná, com destaque para o uso de drogas injetáveis, transfusão sanguínea, drogas inaláveis/crack, tatuagem/piercing e tratamento cirúrgico. A faixa etária e o nível de escolaridade revelaram-se como fatores de proteção. Esses achados responderam ao objetivo proposto e evidenciaram padrões epidemiológicos relevantes para fortalecer ações previstas no Programa Brasil Saudável. Isso contribui para que se identifiquem grupos prioritários e se direcionem estratégias de prevenção, diagnóstico precoce e cuidado contínuo.
